# Characterization of B Cell Responses in Rainbow Trout (*Oncorhynchus Mykiss*) Affected by Red Mark Syndrome

**DOI:** 10.1002/advs.202503047

**Published:** 2025-06-25

**Authors:** J Germán Herranz‐Jusdado, Samuel Vicente‐Gil, Esther Morel, Pablo Jiménez‐Barrios, Rocío Simón, Pedro Perdiguero, Diana Martín, Marta Vargas‐Ramírez, Niels Lorenzen, Jacob Günther Schmidt, Carolina Tafalla

**Affiliations:** ^1^ Skretting Aquaculture Innovation Stavanger 4016 Norway; ^2^ Fish Immunology and Pathology Group, Biotechnology Department, National Institute for Agricultural and Food Research and Technology (INIA) Spanish Research Council (CSIC) Madrid 28040 Spain; ^3^ Animal Health Research Center (CISA), National Institute for Agricultural and Food Research and Technology (INIA) Spanish Research Council (CSIC) Valdeolmos Madrid 28130 Spain; ^4^ Department of Genetics, Physiology and Microbiology, Faculty of Biological Sciences Complutense University of Madrid (UCM) Madrid 28040 Spain; ^5^ Section for Fish and Shellfish Diseases, National Institute of Aquatic Resources Technical University of Denmark Kgs. Lyngby 2800 Denmark

**Keywords:** B cells, IgM, midichloria‐like organism (MLO), plasma cells, red mark syndrome, skin, strawberry disease

## Abstract

Red mark syndrome (RMS) is a disease affecting rainbow trout. Although the precise etiology of this disease is still under debate, a Midichloria‐like organism (MLO) is suspected as the triggering agent. RMS provokes characteristic skin lesions. Previous investigations pointed to a local immune response characterized by a B cell influx. To elaborate on these findings, here, this study extensively characterizes systemic and local B cell responses in RMS‐affected fish. The local influx of IgM+ B cells to the skin lesions is confirmed, and a differentiation of these B cells to plasma‐like cells is demonstrated. IgM repertoires suggested a polyclonal activation of local B cells and some trafficking between skin and head kidney. Finally, the fact that transcripts of the gene coding for MLO 16S rRNA are found in sorted skin IgM^+^ B cells and non‐IgM^+^ skin leukocytes reveals the capacity of this intracellular bacterium to be internalized by B cells, where it may possibly directly interfere with intracellular pathways. The data significantly advances the knowledge on RMS and provides another example of a fish pathology in which fish B cells seem pivotal, possibly because of the specific ways in which teleost B cells intrinsically sense and respond to pathogens.

## Graphical abstract

This study extensively characterizes the B cell response of rainbow trout (*Oncorhynchus mykiss*) in response to red mark syndrome (RMS). This disease, suspected to be caused by a *Midichloria*‐like organism (MLO), induces skin lesions in the infected animals, characterized by a massive influx of IgM^+^ B cells. The data significantly advances the knowledge on RMS.

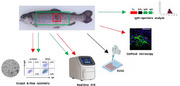

## 1 Introduction

Red mark syndrome (RMS) is a skin condition that affects farmed rainbow trout (*Oncorhynchus mykiss*). RMS was first described in the late 1960s in the USA,^[^ ^1^ ^]^ being initially designated as strawberry disease. Later on, it also appeared in European farms, including the UK.^[^ ^2^ ^]^ Nowadays, outbreaks are spread throughout various continents and affect rainbow trout farms in the UK, Europe, Middle East, America, and Asia,^[^ ^3^, ^4^, ^5^, ^6^ ^]^ with increasing cases every year. RMS is characterized by focal or multifocal, large red skin lesions, typically affecting market‐sized fish.^[^ ^7^ ^]^ The presence of RMS does not appear to affect appetite or growth to a great extent but has a negative impact on the appearance of the fish, resulting in important economic losses for farmers due to downgraded fish products.^[^ ^8^ ^]^ There is no or low mortality associated with RMS, but morbidity can go up to 60%.^[^ ^9^, ^10^ ^]^ Hence, the disease is a challenge for the rainbow trout industry, resulting in important financial setbacks. Although the etiology of RMS is still controversial, multiple recent studies point to a *Midichloria*‐like organism (MLO), a bacterium from the Midichloriaceae family, within the order Rickettsiales, as the causative agent of RMS.^[^ ^10^, ^11^, ^12^, ^13^, ^14^ ^]^ All known species within this order are obligate intracellular organisms, and this is also thought to be the case for the RMS‐associated MLO.^[^ ^15^ ^]^ Although pathology appears to be exclusively associated with the skin, where MLO abundance correlates with RMS pathology,^[^ ^12^ ^]^ MLO can also be found at lower levels in other tissues, such as heart, liver, spleen, intestine, or kidney.^[^ ^10^ ^]^ The first member of the family *Candidatus* Midichloriaceae was isolated from the hard tick, *Ixodes ricinus*.^[^ ^16^ ^]^ However, a number of MLO have since been discovered and partially sequenced, and most of these are from aquatic protists or other invertebrates, where the MLOs are thought to form a symbiotic relationship.^[^ ^17^ ^]^ There are also indications that this may be the case for the MLO associated with RMS.^[^ ^18^ ^]^ However, apart from the RMS‐associated MLO, no other MLOs are known to cause pathology. In addition, no MLOs have been successfully cultured. From the RMS‐MLO, only the gene coding for 16S rRNA has been sequenced. Thus, any functional properties of the bacterium can only be extrapolated from related bacteria. For example, members of the Midichloriaceae are usually flagellated, and they can occur either cytosolic or in vacuoles in host cells.^[^ ^17^ ^]^

Mucosal surfaces are the first line of defense against environmental threats, not only acting as a physical barrier, but also having an important immunological function, through the presence of a variety of immune elements. Due to its lack of keratinization, the skin of teleost fish possesses living epithelial cells in direct contact with the external aquatic environment. This epidermal layer also includes goblet cells that secrete mucus to the outer surface of the epidermis. This mucus provides the first barrier against pathogens.^[^ ^19^ ^]^ In addition, the skin mucosa contains scattered immune cells, including innate immune cells such as mast cells, dendritic cells, or macrophages, as well as adaptive immune cells, i.e., T and B cells.^[^ ^20^ ^]^

It must be taken into account that teleost fish only express IgM, IgD, and the teleost‐specific IgT.^[^ ^21^ ^]^ As in mammals, the main B cell subset in systemic immune compartments is composed of cells that co‐express IgM and IgD on the surface.^[^ ^22^, ^23^ ^]^ Upon antigen encounter, these cells start a differentiation process toward plasmablasts and eventually plasma cells, during which they lose surface IgD.^[^ ^22^ ^]^ Additionally, some B cells, especially found in mucosal surfaces such as skin, gills, or intestine, lose IgM and become IgD^+^IgM^−^ cells, which also have a plasmablast/plasma cell profile.^[^ ^24^, ^25^ ^]^ Therefore, B cells exclusively expressing IgM (IgM^+^IgD^−^ B cells) or IgD (IgD^+^IgM^−^ B cells) constitute the vast majority of cells from this B cell lineage in skin.^[^ ^25^ ^]^ Finally, IgT‐expressing cells constitute an exclusive lineage of B cells, independent of IgM/D cells. Because IgT^+^ B cells are more abundant in mucosal surfaces and IgT responses are dominant in these surfaces in response to some pathogens, IgT has been suggested to be a mucosally dedicated Ig.^[^ ^26^, ^27^, ^28^ ^]^ Nonetheless, nowadays this concept seems less stringent, as IgT responses outside mucosal compartments are being revealed^[^ ^29^, ^30^, ^31^, ^32^ ^]^ and conversely mucosal Ig responses not involving IgT have also been reported.^[^ ^31^, ^33^, ^34^ ^]^

Gene transcriptional analyses and immunohistochemical studies previously undertaken in RMS‐affected rainbow trout pointed to an important role of IgM, and in some cases IgD, in skin lesions,^[^ ^13^, ^33^ ^]^ yet many aspects of this B cell response are still unknown. In contrast, IgT transcription and IgT^+^ cell presence in the skin changed very little at the time points in which skin lesions peaked.^[^ ^33^ ^]^ Hence, with this study, we aimed at more deeply characterizing the local IgM response in RMS‐affected skin. To this end, we infected fish with MLO by co‐habitation following an established model.^[^ ^33^ ^]^ The ratio of seeders to cohabitants was kept low to reduce infection levels and obtain cohabitants with different levels of pathology, including fish with no observable pathology. We sampled fish around the time of peak pathology in the earliest responding cohabitants, thereby obtaining groups of fish with severe pathology (RMS++), mild pathology (RMS+), and no pathology (RMS–) in addition to non‐infected controls. We analyzed IgM responses in these fish using a wide range of immunological techniques including transcriptional studies, ELISpot, ELISA, confocal microscopy, and flow cytometry analysis, including cell sorting. Finally, an IgM repertoire analysis was carried out in the skin and head kidney by next generation sequencing (NGS) to decipher how IgM was responding to the infection and whether there was some B cell trafficking between the skin and the head kidney during disease progression. The results obtained provide novel evidence of yet another fish disease in which IgM^+^ B cells seem to be involved in the development of pathology through an exacerbated response, as it is the case for those provoked by different myxozoan pathogens.^[^ ^30^, ^35^ ^]^ The information generated will help understand better how this uniquely pathogenic MLO provokes RMS, information that will be of relevance for the design of novel immunomodulatory strategies that can reduce the impact of the disease.

## 2 Results

### 2.1 IgM^+^IgD^−^ B Cells Drastically Increase in the Skin of Rainbow Trout with Severe RMS Symptomatology

**Figure 1:**
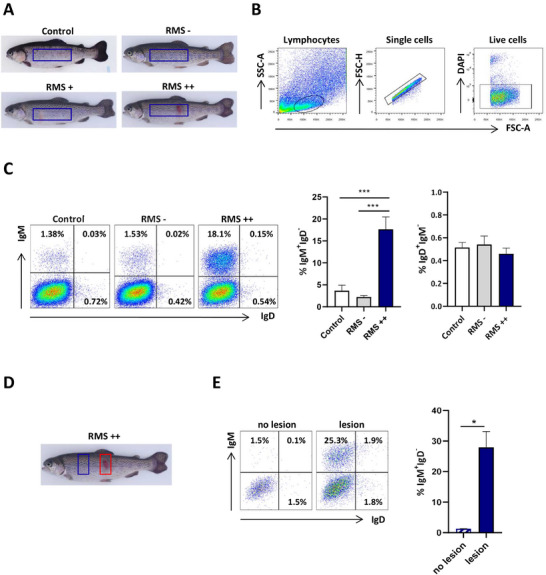
MLO provokes a massive increase of IgM^+^IgD^−^ B cells in the skin. Skin leukocytes were isolated from rainbow trout infected with MLO by co‐habitation, differentiating fish with clear external severe lesions (RMS++), fish with mild lesions (RMS+), and those with no apparent RMS‐like skin pathology (RMS–). Skin leukocytes were also isolated from control non‐infected fish (Control). A) Representative images show a fish from each category in which the sampled skin area is indicated by a blue rectangle. B) Gating strategy for the flow cytometry analysis conducted in this work with skin leukocytes from control, RMS– and RMS++ fish (related to Figures 1C,E, 4A,B, and 5). Lymphoid cells were first gated by FSC/SSC profile. The FSC‐H/FSC‐A profile within the lymphoid gate indicated selected singlets. DAPI negative cells among singlets were gated to select live cells. C) Representative dot plots obtained after staining skin leukocytes isolated from control, RMS– and RMS++ trout with anti‐IgM and anti‐IgD mAbs. Graphs showing the percentage of IgM^+^IgD^−^ and IgD^+^IgM^−^ B cells (mean + SEM; n = 7 independent fish) are also included. Statistical differences among groups were evaluated by an unpaired two‐tailed Student's *t* test when data were normally distributed, whereas non‐normally distributed data were analyzed with the nonparametric Mann‐Whitney test. D) Representative photo of a fish in the peak of the disease (RMS++) indicating skin areas with (red) or without (blue) RMS lesions sampled for the analysis presented in Figure 1E. E) Graph showing the percentage of IgM^+^IgD^−^ B cells (mean + SEM; n = 3 independent fish) in isolated skin leukocytes from areas indicated in **D**, along with representative dot plots. Statistical differences between percentages of cells in lesion‐containing and non‐injured skin were evaluated by a paired two‐tailed Student's *t* test. Asterisks denote significantly different values among groups as indicated (*p < 0.05 and ***p < 0.001).

To verify whether skin B cells were involved in the local immune response to RMS, we isolated skin leukocytes from fish infected by co‐habitation, as well as from control mock‐infected fish (control). Within infected fish, we differentiated between fish with no symptomatology (RMS–), fish with mild lesions (RMS+), and fish with severe lesions (RMS++) (**Figure** **1**A). To study the presence of different B cell populations in isolated skin leukocytes by flow cytometry we exclusively focused on control, RMS – and RMS++ fish, using a big skin fragment to isolate leukocytes as indicated in Figure 1A and gating the cells as indicated in Figure 1B. As previously described,^[^ ^25^ ^]^ the percentages of IgM^+^IgD^+^ B cells found in the skin of control fish were almost non‐existent (≈0.03%), while percentages of IgM^+^IgD^−^ and IgD^+^IgM^−^ B cell populations were higher (≈3.2% and ≈0.52%). Although these percentages were not significantly altered in infected fish with no symptomatology (RMS–), the percentage of IgM^+^IgD^−^ B cells in isolated skin leukocytes from RMS++ fish was drastically augmented (≈18%) in comparison to both RMS – or control fish (Figure 1C). In contrast, no significant differences were observed in the percentages of IgD^+^IgM^−^ B cells among groups, which were ≈0.5% in all cases (Figure 1C). Finally, we wanted to determine whether the increase in IgM^+^IgD^−^ B cells in the skin of RMS++ fish was distributed throughout the entire skin or specifically focused in the lesions. For this, we independently collected sections from skin containing lesions and uninjured skin of RMS++ fish to isolate leukocytes as indicated in Figure 1D. Although the number of leukocytes obtained from these samples was small, it was sufficient to determine the percentage of IgM^+^IgD^−^ B cells by flow cytometry, verifying that the significant increase in IgM^+^IgD^−^ B cells in the skin of RMS‐affected fish is specifically located within the lesions (Figure 1E).

### 2.2 Transcriptional Profile of Rainbow Trout Skin Affected by RMS

**Figure 2:**
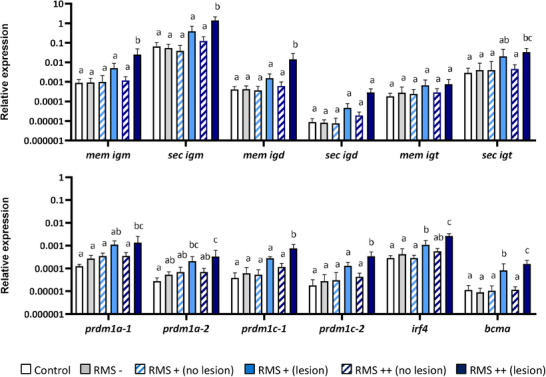
Transcription of genes related to B cell differentiation are differentially regulated in RMS lesions of RMS‐affected fish. Skin samples were collected from rainbow trout infected with MLO by co‐habitation differentiating among RMS–, RMS+, and RMS++, as described in the legend of Figure 1. Samples from non‐infected controls were also included. In the case of RMS+ and RMS++ fish, skin samples were collected from lesions as well as from skin sections without a lesion. RNA was isolated from all skin samples and used to determine the levels of transcription of different genes related to B cell function by real time PCR. These included the membrane (mem) and secreted (sec) forms of IgM, IgD and IgT, the four genes that code for homologues of Blimp1 (*prdm1* genes), *irf4*, and *bcma*. Data are shown as the mean relative gene expression normalized to the transcription levels of the housekeeping gene *b‐actin* + SD (n = 6–7 independent fish for each group). Statistical differences among groups were evaluated by ANOVA. Different lower‐case letters indicate significant differences among groups (*p* < 0.05).

To gain some further insights into the local B cell response of fish affected by RMS, we next studied the levels of transcription of a range of genes associated with B cell function in the skin of infected and non‐infected controls. For this purpose, we collected skin samples from all the fish groups, and in the case of RMS+ and RMS++ fish, we differentiated between lesions and areas of the skin without lesions. As shown in **Figure** **2**, significant increases in the levels of transcription of the membrane and secreted form of IgM, membrane IgD, and secreted IgT in the lesion areas of RMS++ fish were observed when compared to the rest of the samples. We also studied the levels of transcription of genes related to B cell differentiation, including the four genes that code for homologues of Blimp1 (*prdm1* genes), *irf4 (interferon regulatory factor 4)*, and *bcma (B cell maturation antigen)*. All of these genes correspond to genes known to be transcriptionally up‐regulated when B cells differentiate to plasma cells in mammals^[^ ^36^ ^]^ and fish.^[^ ^37^ ^]^ All of them were transcriptionally up‐regulated in the lesions of RMS++ fish, in comparison to control or RMS – skin, and even in comparison to skin with no lesions from RMS++ fish (Figure 2). In the case of *irf4* and *bcma*, the levels of transcription observed in lesion areas of RMS+ fish were also significantly higher than those of RMS – and control fish (Figure 2).

### 2.3 Systemic B Cell Response of RMS‐Affected Rainbow Trout

**Figure 3:**
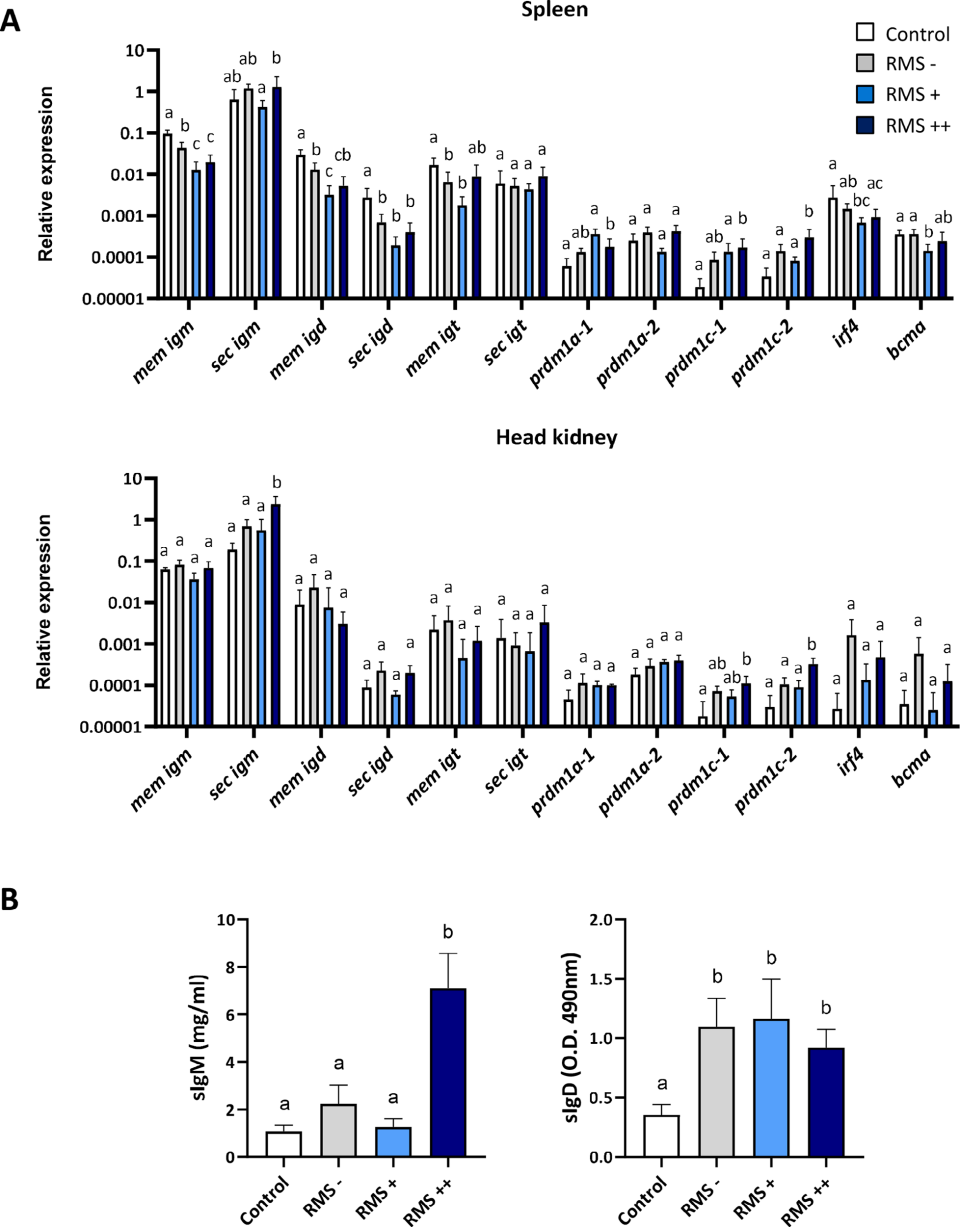
MLO provokes a systemic regulation of B cell function. Spleen, head kidney, and blood samples were collected from rainbow trout infected with MLO by co‐habitation differentiating among RMS–, RMS+ and RMS++, as described in the legend of Figure 1. Samples from mock‐infected controls were also included. RNA was extracted from spleen and head kidney samples and used to determine the levels of transcription of different genes related to B cell function by real time PCR. These included the membrane (mem) and secreted (sec) forms of IgM, IgD, and IgT, the four genes that code for homologues of Blimp1 (*prdm1* genes), *irf4*, and *bcma*. A) Data are shown as the mean relative gene expression normalized to the transcription levels of the housekeeping gene *b‐actin* + SD (n = 7 independent fish for spleen; n = 3 independent fish for head kidney). B) Serum was collected from peripheral blood samples and used to estimate the amount of secreted IgM and IgD by ELISA. Graphs show mean concentration of IgM or the mean absorbance values at 490 nm for IgD (mean + SEM, n = 7 independent fish). Statistical differences among groups were evaluated by ANOVA. Different lower‐case letters indicate significant differences among groups (*p* < 0.05).

To assess whether there is a systemic response to the disease, we also analyzed the transcriptional profile of the different genes related to B cell function in the spleen and head kidney of infected and non‐infected fish. The head kidney is the main hematopoietic tissue in teleost fish as well as the site for B cell maturation and a niche for long‐term maintenance of plasma‐like cells.^[^ ^38^, ^39^ ^]^ As shown in **Figure** **3**A, no genes were transcriptionally up‐regulated in the spleen of fish with RMS symptomatology when compared to infected fish with no symptomatology. On the other hand, the transcription of membrane IgM was significantly down‐regulated in the spleen of RMS+ and RMS++ fish when compared to RMS– and control fish (Figure 3A). Some additional genes were significantly altered in the spleen of RMS++ fish, but only when compared to control fish. These included the membrane and secreted form of IgD (down‐regulated) and *prdm1a‐1*, *prdm1c‐1*, and *prdm1c‐2* (up‐regulated) (Figure 3A). A few genes were also transcriptionally regulated in the head kidney in response to RMS. In this case, the levels of transcription of secreted IgM, *prdm1c‐1*, and *prdm1c‐2* observed in the head kidney of RMS++ fish were also significantly higher than those in the head kidney of control fish (Figure 3A).

To further evaluate the systemic effects of RMS, we also determined IgM and IgD titers in the serum of infected and non‐infected individuals. As shown in Figure 3B, IgM titers in infected fish with no or reduced symptomatology were not significantly different than those of control mock‐infected fish, while IgM drastically increased in the serum of RMS++ fish. In contrast, IgD levels in serum were significantly increased in all infected groups, regardless of the RMS pathology shown (Figure 3B).

### 2.4 Skin IgM^+^IgD^−^ B Cells from Rainbow Trout with RMS are further Differentiated to a Plasma‐Like Profile

**Figure 4:**
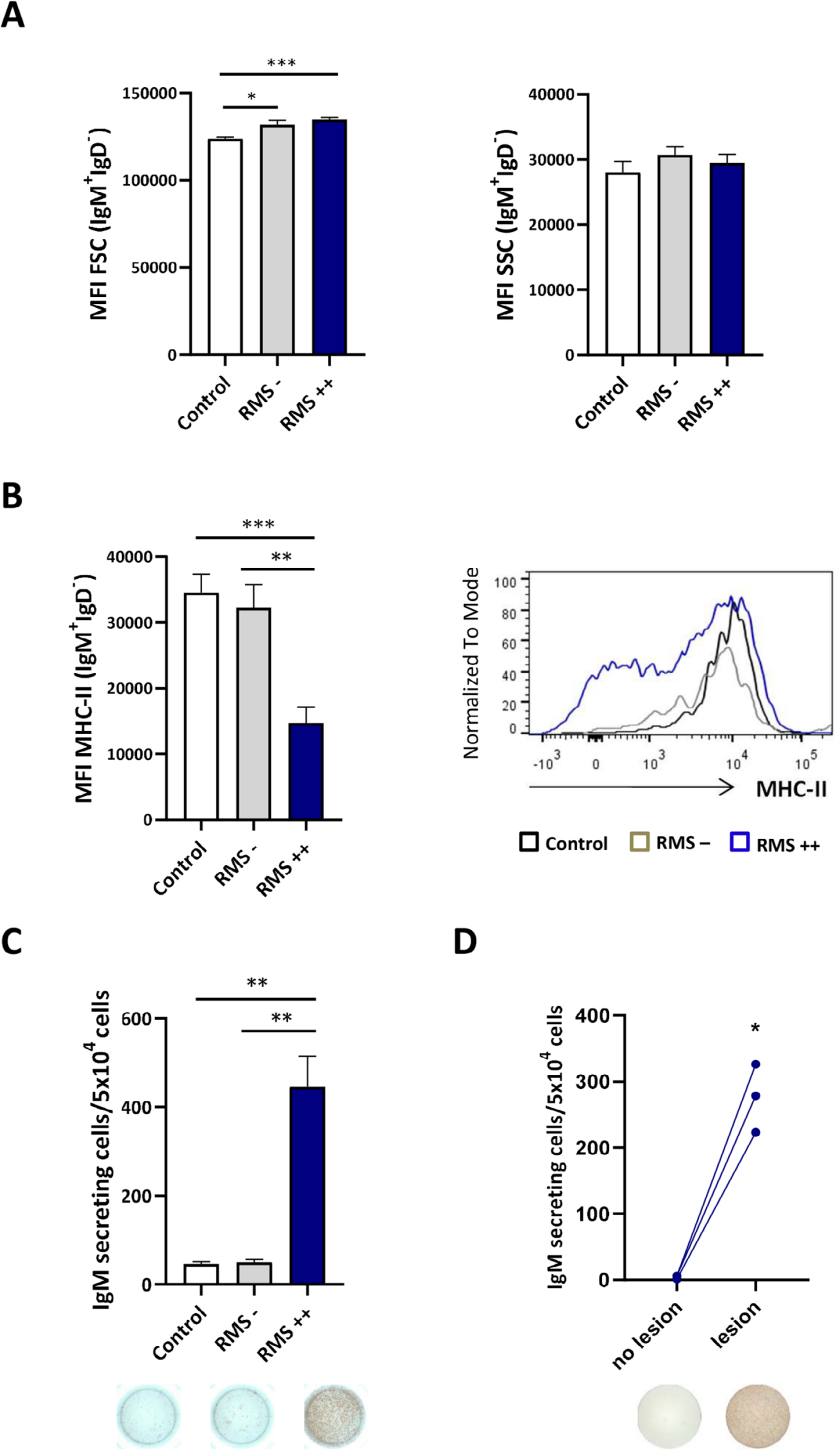
IgM^+^IgD^−^ B cells in the skin of RMS‐affected rainbow trout have a more pronounced plasma‐like profile. Skin leukocytes were isolated from rainbow trout infected with MLO by co‐habitation differentiating among RMS– and RMS++, as described in the legend of Figure 1. Skin leukocytes were also obtained from non‐infected controls. Isolated leukocytes were stained with specific anti‐IgM, anti‐IgD, and anti‐MHC‐II mAbs. A) Graphs showing the size (referred to as forward scatter, FSC) and cell complexity (referred to as side scatter, SSC) of skin IgM^+^IgD^−^ B cells from control, RMS – and RMS++ fish (mean + SEM, n = 7 independent fish). B) Graph showing the mean fluorescence intensity (MFI) values of MHC II on skin IgM^+^IgD^−^ B cells from control, RMS–, and RMS++ fish (mean + SEM, n = 7 independent fish) are shown along with a representative histogram. C,D) Skin leukocytes were isolated from control, RMS–, RMS++ fish, and used to determine the number of cells secreting IgM by ELISpot. For that, cells were counted and plated on the day of isolation in ELISpot plates pre‐coated with anti‐IgM mAb. C) Graph shows mean number of IgM‐secreting cells per 5 × 10^4^ cells + SEM (n = 7 independent fish) in isolated leukocytes from control, RMS –, and RMS++ fish. D) For RMS++ fish, the number of IgM‐secreting cells was calculated in leukocytes obtained from skin areas with or without lesions. Graph shows mean number of IgM‐secreting cells per 5 × 10^4^ cells (n = 3). Statistical differences among groups were evaluated by an unpaired nonparametric Mann‐Whitney test (A‐C) or a paired two‐tailed Student's *t* test (D). Asterisks denote significantly different values among groups as indicated (*p < 0.05, **p < 0.01, ***p < 0.001).

The transcriptional profile of the skin in RMS‐affected trout strongly suggested that B cells in lesions were more differentiated toward a plasma‐like profile than the B cells in asymptomatic skin from the same fish or B cells from asymptomatic or control fish. We confirmed this at a cellular level, through both flow cytometry and ELISpot. As occurs in mammals, when fish B cells differentiate toward a plasmablast/plasma cell profile they increase their size.^[^ ^22^, ^37^ ^]^ Interestingly, when we compared the size of IgM^+^IgD^−^ B cells in skin of control, RMS – and RMS++ fish, we observed that those of infected fish (either RMS – or RMS++) were significantly bigger (referred to as forward scatter, FSC) than those of control fish (**Figure** **4**A). In contrast, no changes in complexity (referred to as side‐scattered, SSC) were detected among skin IgM^+^IgD^−^ B cells from the different fish (Figure 4A). Often changes in complexity are not observed after fish B cell differentiation.^[^ ^37^ ^]^ Finally, the levels of surface MHC II were significantly reduced in IgM^+^IgD^−^ B cells from RMS++ fish, in comparison to the levels observed in both control fish and infected fish with no symptomatology (Figure 4B). Reduction in MHC II levels is one of the hallmarks of B cell differentiation in mammals.^[^ ^40^ ^]^ As an indication of the B cell differentiation toward plasma‐like cells, we also studied the number of IgM‐secreting cells in isolated skin leukocytes by ELISpot. As shown in Figure 4C, the number of IgM‐secreting cells was drastically higher in isolated leukocytes obtained from the skin of RMS++ in comparison to the number of cells detected in control fish or fish with no symptomatology (RMS‐). In the case of RMS++ fish, this increase in the number of IgM‐secreting cells was significantly higher in cell cultures obtained from RMS lesions compared to those obtained from unaffected skin (Figure 4D).

### 2.5 Skin IgM^+^ B Cells from Rainbow Trout with Severe RMS Symptomatology Have a Differentiated Plasmablast Transcriptional Profile

**Figure 5:**
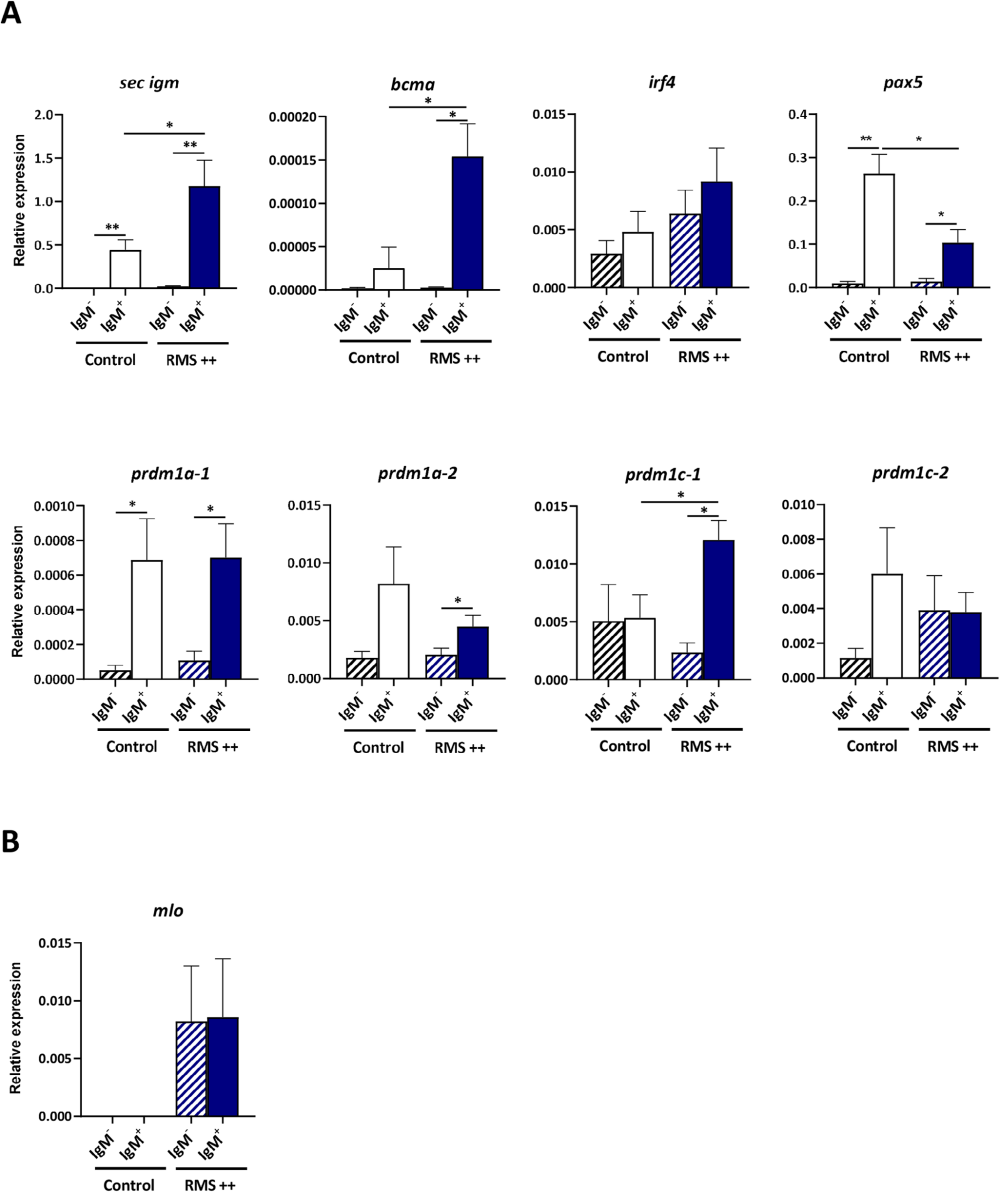
Transcriptional profile of sorted IgM^+^ and IgM^−^ B cells from the skin of rainbow trout with RMS. Skin leukocytes were isolated from rainbow trout infected with MLO by co‐habitation, showing severe signs of disease (RMS++) and from non‐infected controls and stained with a specific anti‐trout IgM mAb. IgM^+^ and IgM^−^ cell populations isolated from both experimental groups were sorted by flow cytometry as described in the Materials and Methods section. A) Graphs showing the levels of transcription of several genes related to B cell function including secreted *igm (sec igm)*, *bcma*, *irf4*, *pax5*, *prdm1a*‐*1*, *prdm1a‐2*, *prdm1c*‐*1*, and *prdm1c‐2* by real‐time PCR. B) These samples were also used to amplify the 16S rRNA gene of MLO. In all cases, results are shown as expression values relative to endogenous control *b‐actin* in the different subpopulations (mean + SEM, n = 5 independent fish). Statistical differences between IgM^+^ and IgM^−^ in Control and RMS++ fish were evaluated by a paired two‐tailed Student's *t* test when data were normally distributed, whereas non‐normally distributed data were analyzed with the non‐parametric Wilcoxon matched‐pairs signed‐rank test. Statistical differences between Control IgM^+^ and RMS++ IgM^+^ samples were evaluated by an unpaired two‐tailed Student's t test when data were normally distributed, whereas non‐normally distributed data were analyzed with the nonparametric Mann‐Whitney test. Asterisks denote significantly different values among groups as indicated (*p < 0.05, **p < 0.01).

To unequivocally confirm that the IgM^+^IgD^−^ B cell subset found in skin from RMS++ fish are more differentiated toward a plasma‐like profile than those of control fish and characterize further this differentiation, we sorted IgM^+^ and IgM^−^ cells from control non‐infected and from RMS++ fish and compared the levels of transcription of secreted *igm*, *bcma*, *irf4, pax5* and the four *prdm1* genes. Within the B cell lineage, *pax5* is expressed from the pre‐B cell through mature B stages and is downregulated during the differentiation to plasma cells.^[^ ^41^ ^]^ Some of these genes were differentially regulated in IgM^+^ compared to IgM^−^ cells regardless of whether the fish were infected or not. For example, secreted *igm*, *pax5*, and *prdm1a‐1* transcription levels were significantly higher in IgM^+^ cells than in IgM^−^ cells from all fish (**Figure** **5**A). When comparing IgM^+^ cell fractions from non‐infected controls and RMS++ fish, the levels of transcription of secreted *igm*, *bcma*, and *prdm1c‐1* were significantly higher in the RMS++ fish (Figure 5A). In contrast, *pax 5* mRNA levels were significantly lower in IgM^+^ B cells from RMS++ fish compared to those of IgM^+^ B cells in control fish (Figure 5A). Thus, the observed differential expression levels are consistent with a further differentiation of IgM^+^ cells toward a plasma‐like profile in RMS++ fish.

### 2.6 The MLO 16S rRNA Gene is Transcribed in Skin Leukocytes from RMS‐Affected Fish

Interestingly, the MLO 16S rRNA gene was transcribed in both IgM^+^ and IgM^−^ skin leukocytes of RMS++ fish but never in those of control non‐infected fish (Figure 5B), providing additional evidence to MLO as the infectious agent triggering the disease and pointing to skin leukocytes as targets for the replication of this intracellular bacterium. In RMS++ fish, the levels of transcription of this gene did not significantly differ between IgM^+^ B cells and IgM^−^ leukocytes (Figure 5B).

### 2.7 Skin IgM^+^ B Cells from Rainbow Trout with RMS Symptomatology are not Proliferating Locally

**Figure 6:**
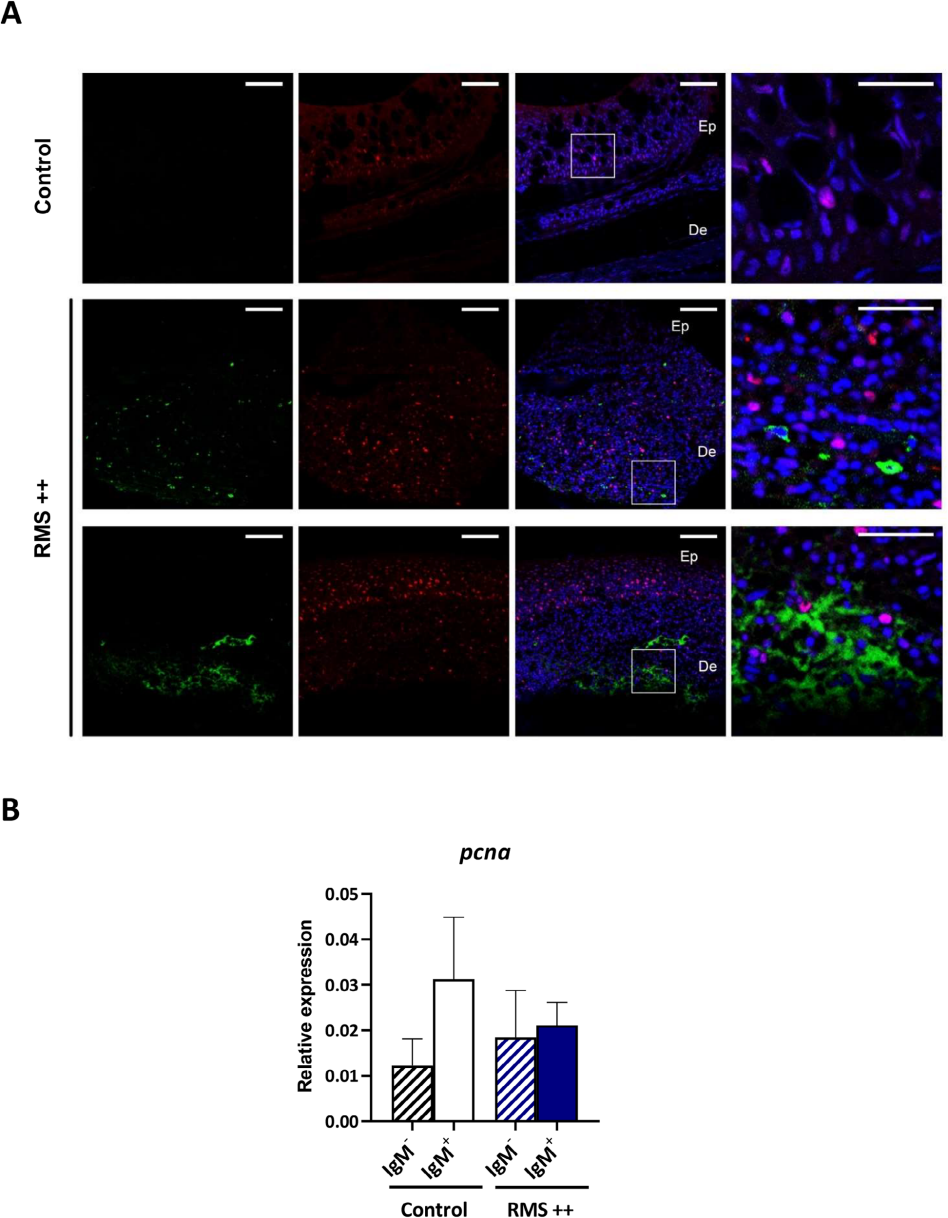
IgM^+^ B cells in the skin of RMS‐affected fish are not significantly proliferating. A) Confocal microscopy images of rainbow trout skin sections obtained from fish experimentally infected with red mark syndrome (RMS++) (lesion site) and from fish that have never been exposed to the pathogen (Control). Sections were labeled with anti‐IgM (green) in combination with anti‐proliferating cell nuclear antigen (PCNA) (red). All sections were also counterstained with DAPI (blue). Representative images of each condition are shown (scale bars = 20 µm) along with representative images (white square) at higher magnification (right; scale bars = 5 µm). Control images are on the top, and RMS++ images from two individual fish are shown in the two lower rows. Note that in non‐proliferating cells, nuclei appear blue whereas they appear violet in proliferating cells. Ep = epidermis; De = dermis. B) Skin leukocytes were isolated from rainbow trout exposed to RMS by co‐habitation, showing severe signs of disease (RMS ++) and from mock‐infected controls and stained with a specific anti‐trout IgM mAb. IgM^+^ and IgM^−^ cell populations isolated from both experimental groups were sorted to determine the levels of transcription of *pcna* gene. Data are shown as relative expression values to endogenous control *b‐actin* in the different subpopulations (mean + SEM, n = 5 independent fish for each group). No statistical differences were found among different groups.

To decipher whether the increased number of IgM^+^ B cells in the skin of RMS‐affected fish was a consequence of a local proliferation of IgM^+^ B cells, we performed confocal analysis staining of skin sections from RMS++ and control fish with a specific anti‐IgM mAb and a mAb against proliferating cell nuclear antigen (PCNA). PCNA expression identifies cells in all active phases of the cell cycle.^[^ ^42^ ^]^ As expected, the number of IgM^+^ B cells in skin sections from rainbow trout with RMS symptomatology were higher than those observed in controls, where only a few scattered cells were visualized (**Figure** **6**A). Additionally, more PCNA^+^ cells were detected in skin from RMS+ fish compared to skin from controls. In the case of RMS++ fish, these PCNA^+^ cells were located both in the dermis and the epidermis, while in control fish the few PCNA^+^ cells seemed to be restricted to the epidermis layer (Figure 6A). However, none of the IgM^+^ B cells identified in RMS‐affected fish were positive for PCNA (Figure 6). In some cases, we detected large areas with a diffuse staining for IgM in skin sections of RMS‐affected fish (Figure 6A, lower panels). These areas were located in the dermis and were inexistent in control mock‐infected fish, suggesting they could be a consequence of the high quantities of secreted IgM produced by IgM secreting cells located in the RMS lesions.

To unequivocally confirm that IgM^+^ B cells detected in skin from RMS‐affected fish were not proliferating locally, we sorted skin IgM^+^ and IgM^−^ cells from control mock‐infected fish and infected fish with severe symptomatology (RMS++) and compared the levels of expression of *pcna* gene among the different subsets. As shown in Figure 6B, the levels of expression of *pcna* were very similar among the different cell subsets and no significant differences were detected in any case, confirming that skin IgM^+^ B cells in RMS++ fish do not have an increased proliferative capacity.

### 2.8 Investigation of Clonal Expansion of IgM in the Skin of Rainbow Trout Affected by RMS

**Figure 7:**
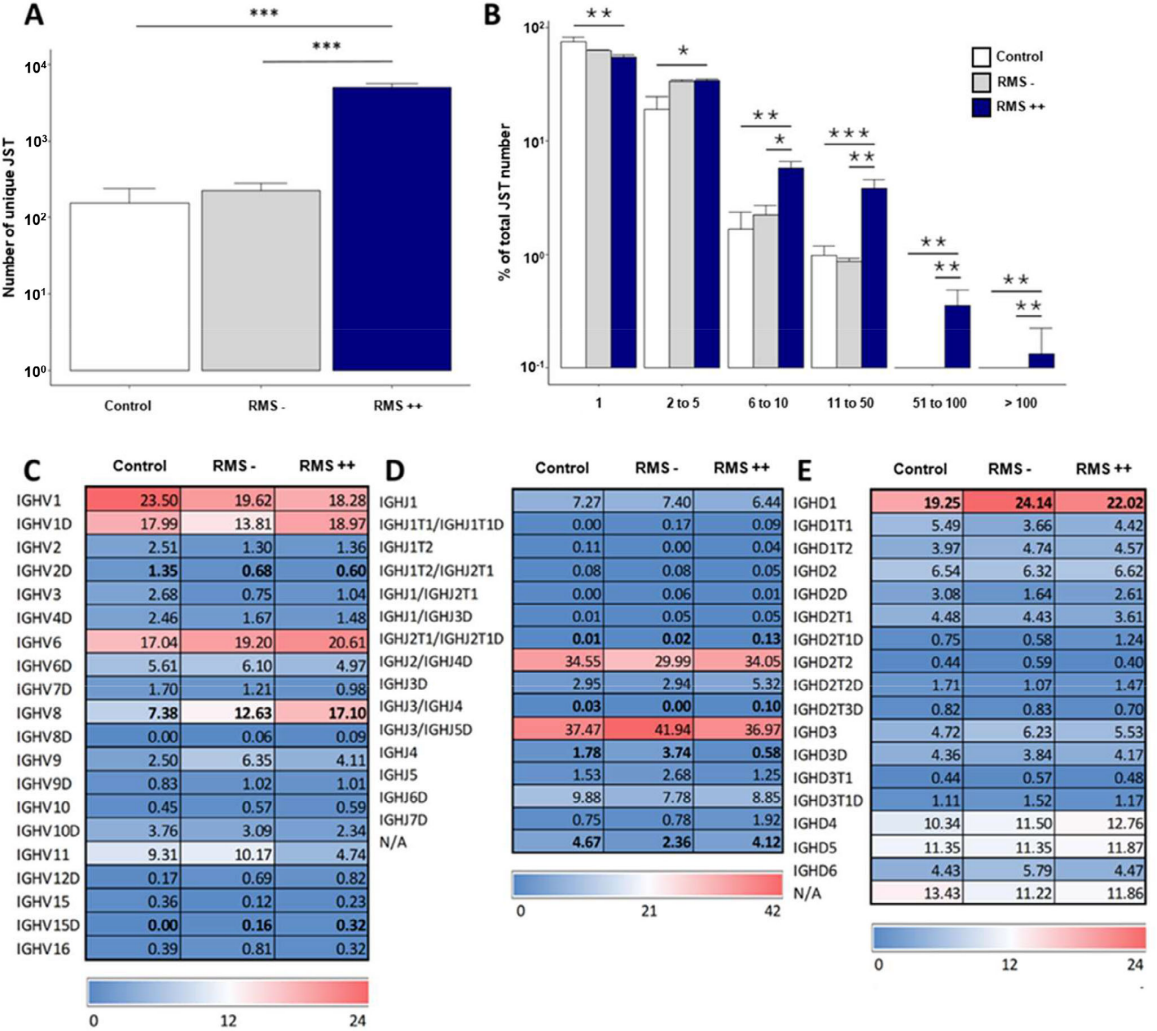
IgM V_H_DJ_H_ gene configuration in the skin during RMS. Skin samples were collected from rainbow trout infected with MLO by co‐habitation differentiating among RMS– and RMS++, as described in the legend of Figure 1. Samples from non‐infected controls were also included. All samples were used to isolate RNA and perform a repertoire analysis of the IgM BCR as indicated in the Materials and Methods section. A) Bar charts show the total number of unique junction sequence types (JSTs) in each group (mean + SD, n = 5). B) Grouped clonal size distribution for IgM across the different groups. Bar charts show the relative frequency of JSTs observed n times in each condition (mean + SD, n = 5). Significant differences among groups were evaluated by ANOVA, followed by a Tukey's HSD post‐hoc test when data were normally distributed, whereas non‐normally distributed data were analyzed with the non‐parametric Kruskal‐Wallis test. Asterisks denote significantly different values among groups as indicated (*p < 0.05, **p < 0.01; ***p < 0.001). C–E) Heatmap representations of usage of differential germline C) Ig V_H_, D) J_H_, and E) D_H_ genes. Each value represents the mean relative frequency observed for each gene reaching at least 0.05% of sequences in one or more conditions (n = 5). Statistical differences among groups were evaluated by ANOVA, for normal samples, or by Kruskal‐Wallis, for no‐parametric samples, and significant ones are highlighted in bold (p < 0.05).

To gain additional insight into the rainbow trout IgM response to RMS, we performed an IgM repertoire analysis in the skin to dissect the heavy chain variable region (V_H_) family usage in control fish as well as in infected fish, distinguishing between fish with no symptomatology (RMS–) and fish with severe lesions (RMS++). As previously described,^[^ ^30^, ^43^ ^]^ unique sequences defined as V(D)J rearrangements associated with a specific CDR3 amino acid sequence were referred to as junction sequence types (JST). Hence, we could confirm that the number of JSTs in the skin of RMS++ fish was ≈100‐fold higher than that of control fish or RMS– fish (**Figure** **7**A), indicating a greater diversity of IgM. To investigate if there was clonal expansion of IgM in response to RMS, we dissected the clonal size distribution of JSTs. It is well established that JSTs detected fewer than 3–5 times in a given tissue correspond to non‐expanded antigen‐naive B cells, whereas JSTs detected more than 50–100 times reflect expanded antigen‐experienced B cell clones, including plasmablasts and plasma cells.^[^ ^24^, ^29^ ^]^ We found that the number of sequences only repeated once were significantly lower in infected fish with symptomatology (RMS++) than in control fish (Figure 7B). On the other hand, the number of sequences that were repeated more than 6 times, and even more than 50–100 times, were significantly higher in the skin of RMS++ when compared to that of control fish or RMS– fish (Figure 7B). These results confirm a clonal expansion of IgM in the skin during development of RMS pathology. To better understand this expansion, we next analyzed the usage of the different V, D and the J segments in each of the IgM sequences detected in the skin.

Some V_H_ families were predominantly used in the skin regardless of the infection, such as IGHV1, IGHV1D, IGHV6, IGHV8 and IGHV11, which all together accounted for 75–79% of the sequences in the skin. When fish were infected, this pattern of V segment usage only changed slightly. Hence, for example, there was a decreased usage of IGHV2D and an increased usage of IGHV8 and IGHV15D (Figure 7C). Concerning J segments, there was a strong preference for IGHJ2/IGHJ4D or IHGJ3/IGHJ5D in the skin, together accounting for 71–72% of all CDR3 sequences (Figure 7D). Again, only a few changes in this pattern were detected in response to the infection. The percentage of sequences with IGHJ2T1/IGHJ2T1D and IGHJ3/IGHJ4 significantly increased in the RMS++ skin, whereas the percentage of sequences with no J segment significantly decreased in the infected fish with no symptomatology (Figure 7D). Also, the percentage of sequences with IGHJ4 significantly increased in the RMS‐ samples and decreased in the RMS++ samples relative to non‐infected controls (Figure 7D). Finally, most IgM sequences in the skin used IGHD1 (19‐24%), followed by IGHD4 (10‐13%) and IGHD5 (≈11.5%), whereas the percentage of sequences lacking IGHD was also high (11‐13.5%) (Figure 7E). The usage of IGHD1 was increased in response to MLO infection, while no other significant changes were detected (Figure 7E).

### 2.9 Comparative Repertoire Analysis of Skin and Head Kidney IgM in RMS++ Fish

**Figure 8:**
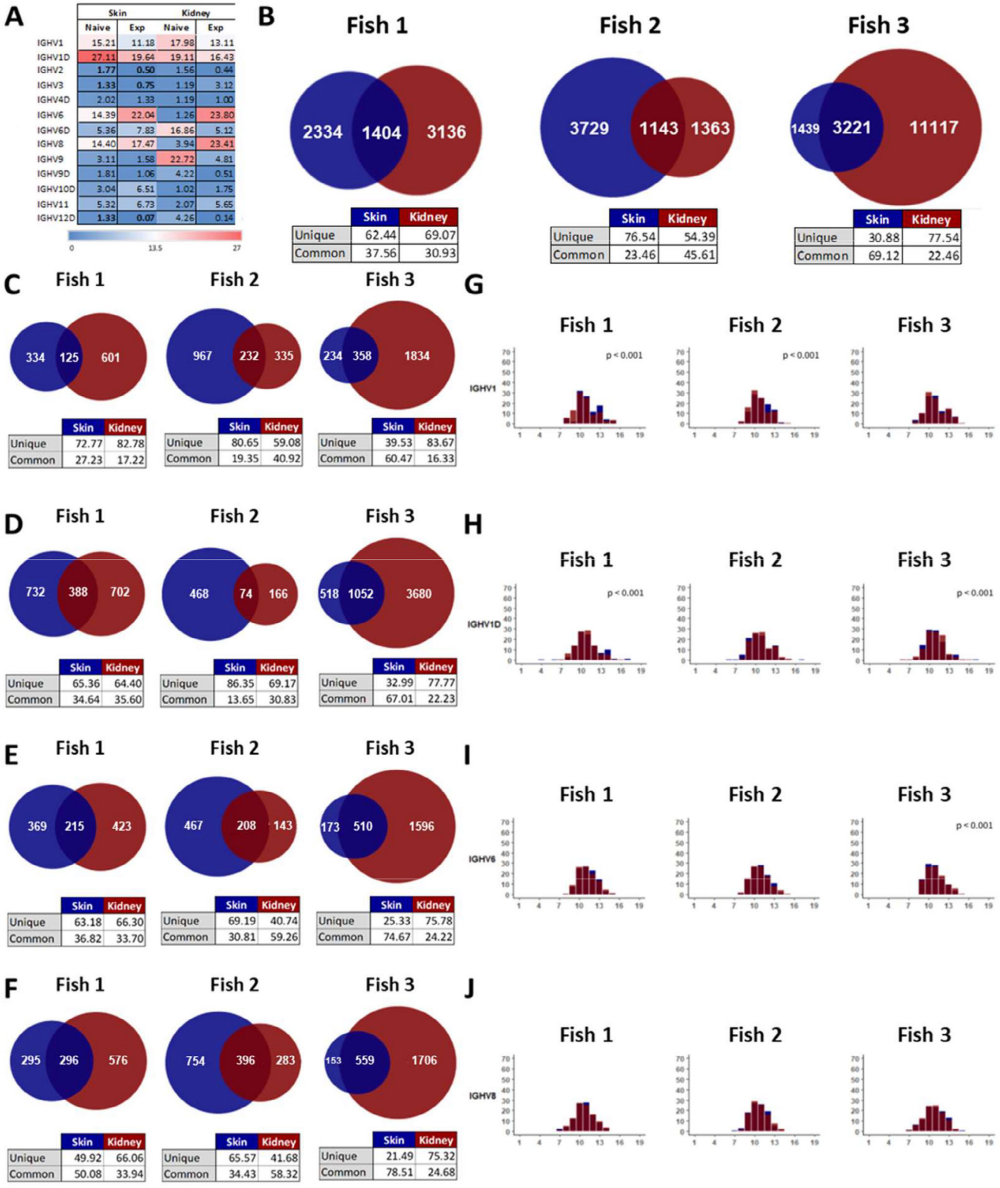
Comparison of skin and head kidney IgM repertoires in RMS++ fish. Three RMS++ fish were selected to perform an additional IgM repertoire analysis in their head kidney. A) Heatmap representation of differential germline Ig V_H_ usage in skin and kidney samples from RMS++ fish among naive (JST 1–5 times) and expanded (Exp; JST > 10 times) IgM sequences. Each value represents the mean (n = 3) relative frequency observed for each gene reaching at least 2.5% of sequences between both conditions. Statistical differences in naive against expanded sequences were detected with a paired two‐tailed Student´s t test and are highlighted in bold (p < 0.05). B) Venn diagrams showing shared CDR3 sequences between the skin (blue) and kidney (brown) samples in fish 1, 2, and 3. The tables below indicate the percentage of unique and shared CDR3 sequences in each sample. C–F) Shared CDR3 sequences between skin (blue) and kidney (brown) this time exclusively selecting the most common V_H_ genes used: C) IGHV1, D) IGHV1D, E) IGHV6, and D) IGHV8. G–J) Overlapping charts showing CDR3 spectratypes of IgM transcripts from sequences corresponding to the most expanded IgM V_H_ families: G) IGHV1, H) IGHV1D, I) IGHV6, and J) IGHV8. Statistical differences between samples from skin and kidney were evaluated by non‐parametric Mann‐Whitney test.

Among the five RMS++ fish in which we studied the skin IgM repertoire, we selected three to perform an IgM repertoire in the head kidney. This compared analysis would provide us with an indication of whether IgM clonotypes were similar in skin and head kidney in these fish, which might suggest some kind of trafficking of IgM^+^ B cells between the two tissues. First, we compared the usage of the different V_H_ segments in skin and head kidney samples from RMS++ fish differentiating between naive (JST 1–5 times) and expanded (Exp; JST > 10 times) IgM sequences. As shown in **Figure** **8**A, the usage of V segments was very similar between naïve and expanded IgM sequences in the skin, with only a few minor significant differences. The pattern of V_H_ usage was very similar in the head kidney, but in this case, although some segments were preferentially used in expanded sequences, the differences were not significant (Figure 8A).

We next compared all CDR3 sequences from skin and head kidney (without differentiating between naïve and expanded sequences) in each fish to identify common sequences. Skin shares 23.5–37.5% of its CDR3 sequences with the kidney in the two of the fish analyzed, while this percentage increased to 69% in the last fish (Figure 8B). These differences seem to be due to large differences in the number of different CDR3 sequences among the three head kidney samples (3100‐11100), while they are not due to differences in the skin, where the number of different CDR3 sequences is similar in all fish (3700‐4600). Additionally, we performed this comparison, but this time dissecting the results from sequences containing the most expressed V_H_ genes (IGHV1, IGHV1D, IGHV6, and IGHV8) (Figure 8C–F). Also in this case, the percentage of shared sequences between the skin and the head kidney was much higher in Fish 3 (Figure 8C–F). Nonetheless, the percentages of shared sequences were slightly lower for IGHV1 (Figure 8C) or IGHV1D (Figure 8D) when compared to the results obtained examining all CDR3 sequences. Interestingly, in the case of IGHV6 (Figure 8E) or IGHV8 (Figure 8F) sequences, the percentage of shared sequences between skin and head kidney is increased in comparison to the results obtained when all CDR3 sequences are included in the analysis, suggesting that it is precisely sequences containing these segments which are preferentially shared. Actually, in the case of IGHV8, the percentage of sequences in the skin that were also found in the head kidney were 50%, 34%, and 79% (Figure 8F).

To further understand how similar the IgM repertoires were in skin and head kidney of the RMS++ fish, we also studied the CDR3 length by spectratyping to estimate the diversity of the Ig gene repertoire in both tissues, focusing on the most expanded V_H_ families (IGHV1, IGHV1D, IGHV6, and IGHV8). As previously established,^[^ ^29^ ^]^ we can determine that B cell clonal selection takes place when the CDR3 length distribution deviates from a bell‐shaped Gaussian distribution. In this case, all distributions seemed to reflect some kind of clonal selection as their distribution differed significantly from a normal distribution in all cases (data not shown). Nonetheless, what was interesting was the high degree of similitude between the spectratyping from skin and that of head kidney (Figure 8G–J), which was even highest in the case of sequences using the IGHV8 segment (spectratypes not significantly different) (Figure 8J), thus indicating a high degree of shared clonotypes between the two tissues in these animals.

## 3 Discussion

In the recent past, a number of studies have revealed that fish B cells retain many innate‐like capacities, phenotypically and functionally resembling to some extent innate mammalian B cell populations such as B1 cells.^[^ ^44^, ^45^, ^46^ ^]^ Mammalian B1 cells are responsible for the early recognition of pathogens, providing a first wave of T cell‐independent responses that include the secretion of large amounts of IgM.^[^ ^47^, ^48^ ^]^ These antibodies, even if of low affinity, are able to partially neutralize pathogen progression until a more specific T cell dependent B2 cell response is mounted.^[^ ^49^ ^]^ Similarly, teleost B cells have been shown to be well‐equipped with a range of innate receptors to help them recognize pathogens,^[^ ^50^ ^]^ have a strong phagocytic and microbicidal activity^[^ ^44^ ^]^ and consequently have been shown to be one of the main players of early inflammatory responses in fish, also helping in the initial clearing of pathogens.^[^ ^51^, ^52^ ^]^ It might be possible that it is precisely due to this different way in which fish B cells sense pathogens that some fish diseases are accompanied by an exacerbated B cell response, being this B cell dysregulation in some cases an important factor in the development of pathology. This is the case in proliferative kidney disease (PKD), a disease that affects trout populations, provoked by the myxozoan parasite *Tetracapsuloides bryosalmonae*.^[^ ^53^ ^]^ The parasite mainly affects the kidney, where it induces a profound alteration of all B cell populations, involving their polyclonal expansion.^[^ ^30^ ^]^ A similar response is provoked by another myxozoan parasite, *Sphaerospora molnari*, that infects common carp.^[^ ^35^ ^]^ This parasite drastically increases the number of circulating B cells, yet this increase is not correlated with a high titer of specific IgMs, again suggesting a polyclonal activation of B cells that leads to hypergammaglobulinemia.^[^ ^35^ ^]^

In the current study, we have demonstrated that RMS lesions are characterized by a drastic influx of IgM^+^ B cells, which also seem to play an important role in the pathogenesis. Similarly to the IgM^+^ B cell populations present in the rainbow trout skin in homeostasis, these cells are IgM^+^IgD^−^ B cells.^[^ ^25^ ^]^ Although some studies have pointed to an increase of IgD in the skin of RMS‐affected rainbow trout,^[^ ^33^ ^]^ in our experiments, although membrane IgD transcription levels were significantly affected in the RMS lesions, this increase was not confirmed by flow cytometry, since the percentage of IgD^+^IgM^−^ B cells remained unaltered. Other studies also reported no local changes in IgD during RMS,^[^ ^13^ ^]^ therefore it might be possible that this Ig is only involved at specific time points or in certain conditions. Nonetheless, the levels of IgD in the sera of infected fish was significantly higher than that of controls, but no differences were observed among RMS–, RMS+, and RMS++ groups. This result reveals that although there seems to be a systemic response of IgD to the infection, there is no correlation of IgD levels with the RMS pathology. In contrast to what occurs with IgD^+^IgM^−^ B cells, the percentage of IgM^+^IgD^−^ B cells drastically increased in the RMS lesions. In homeostasis, skin IgM^+^IgD^−^ B cells have a phenotype of cells that have already started a differentiation program toward plasmablasts or even plasma‐like cells, when compared to blood naïve B cells.^[^ ^25^ ^]^ However, we have seen that in RMS these cells can differentiate even further, up‐regulating the transcription of genes related to B cell differentiation, augmenting their size and increasing their IgM secreting capacity. The fact that skin B cells can further differentiate in response to a pathogenic encounter has also been demonstrated in rainbow trout in response to *Yersinia ruckeri*.^[^ ^54^ ^]^ In fish with a severe RMS pathology, skin IgM^+^IgD^−^ B cells also significantly decreased their levels of surface MHC II expression. It must be taken into account that in homeostasis, despite their differentiated phenotype, skin IgM^+^IgD^−^ B cells retain surface MHC II at levels similar or even higher than those of blood naïve B cells.^[^ ^25^ ^]^ This is also true for other rainbow trout plasma‐like populations such as those found in the adipose tissue.^[^ ^23^ ^]^ Nonetheless, the reduction of the antigen presenting machinery is a characteristic feature of switched plasma cells in mammals,^[^ ^40^ ^]^ and therefore, the fact that MHC II surface levels are significantly reduced in RMS++ fish suggests that these cells undergo a profound differentiation, possibly focusing the cellular machinery to IgM secretion and reducing all other functionalities.

Having confirmed the increased number of IgM^+^IgD^−^ B cells in the RMS lesions, we wanted to determine whether this increase was due to a local proliferation of residing skin B cells or instead a consequence of IgM^+^ B cell migration from another immune organ. For this, we first determined the proliferating capacity of skin IgM^+^ B cells. Unexpectedly, the proliferative capacity of IgM^+^ B cells did not increase in the RMS lesions, as confirmed by both confocal microscopy and real‐time PCR. On the other hand, proliferation of non‐IgM cells was extensive in the lesions. Whether these proliferating cells are epithelial cells aiming to repair the lesion or also involve immune cells is something that should be investigated in future studies. Nonetheless, the results obtained suggest that IgM^+^ B cells somehow are recruited into the RMS lesions from other immune organs, possibly already in a differentiated state, since B cell differentiation usually involves cell proliferation. The fish kidney, especially in its more anterior region (the head kidney) constitutes the main hematopoietic tissue and a site for B cell differentiation.^[^ ^38^ ^]^ Furthermore, the posterior part of the kidney has been shown to be a niche for long‐term storage of plasmablasts and plasma‐like cells.^[^ ^39^ ^]^ For this reason, we investigated if there might be some kind of trafficking between the skin and the head kidney in RMS by studying their IgM repertoire in three severely RMS‐affected fish. The first thing we noticed was that the usage of V segments was very similar in the IgM BCR repertoires of skin and head kidney in these fish. Additionally, the CDR3 length distributions were also quite similar in these two tissues, especially in the case of BCR sequences that used the IGHV8 segment. Finally, although there was a large variability among the three fish studied, the percentage of shared BCR sequences between skin and kidney in these fish was also quite high. Remarkably, it was in the case of BCR sequences that used the IGHV8 segment that the shared percentages were higher. Hence, the fact that there is a high percentage of shared IgM clonotypes between the skin and the kidney in these fish strongly suggests some kind of trafficking between these two tissues in RMS. Notably, the percentages of shared clonotypes between the skin and the spleen was much lower (data not shown). On the other hand, transcriptional changes in head kidney with regards to genes involved in the B cell response were minor. The same was the case for the spleen, and although some significant differences were observed among groups, these did not seem to be specifically linked to fish with an increased symptomatology, since in most cases, the levels of expression observed in RMS++ fish were only significantly different when compared with non‐infected controls and not with RMS– fish. Nonetheless, RMS++ fish do highly increase serum IgM titers suggesting a systemic alteration of the B cell response. It has to be taken into account that if plasma‐like cells are circulating between the kidney and the skin, they would have to do this through the blood, therefore, the high IgM titers in blood could be the consequence of an increased number of circulating IgM‐secreting cells. This is something that should be confirmed in future studies.

Despite the high titers of total IgM in the blood of RMS++ fish, we are not sure that these IgMs are in fact specific against MLO. Given that the bacterium has never been isolated and cultured, it is difficult to establish a protocol to analyze MLO‐specific IgM titers. Nonetheless, the fact that RMS – have significantly lower IgM titers in blood than those of RMS++ fish point to this induced IgM production as unable to control disease progression, possibly being more involved in the pathogenesis of the disease than in its resolution. Furthermore, when we compared the repertoire of IgM among control, RMS – and RMS++ fish, we observed that the pattern of V, D, J segment usage was not extensively modified in infected fish, with significant differences mostly observed in segments that were barely used by skin B cells. Hence, the skin IgM repertoire seemed almost unaffected by the infection with MLO. A very limited difference in repertoire was also observed when comparing skin and head kidney from RMS++ fish. Therefore, although the CDR3 spectratyping analysis of the BCRs in the skin and head kidney of RMS++ shows some kind of perturbation, we can affirm that RMS does not involve a strong clonal selection of B cells, which suggests an inefficient IgM response, unable to control the development of the disease. This situation is similar to that observed in the PKD, where infected fish increased the usage of many V segments, without a particular focus on a specific BCR.^[^ ^30^ ^]^

We found MLO 16S rRNA transcripts within the IgM^+^ B cells of RMS‐affected fish, as well as in other leukocyte subsets from the skin, but never from non‐infected animals. These results add further evidence of MLO being the etiological agent of RMS, being this the only organism of the Midichloriaceae family known to cause pathology.^[^ ^16^ ^]^ These results also suggest that the MLO internalization in these leukocyte subsets is mediating at least partially their functional alteration. Interestingly, macrophages/monocytes and endothelial cells are typical target cells for obligate intracellular bacteria,^[^ ^55^ ^]^ whereas B cells are very uncommon target cells for these type of pathogens. The fact that an obligate intracellular bacteria such as MLO is internalized in rainbow trout B cells may be a consequence of the high phagocytic capacity of teleost B cells relative to mammalian B cells,^[^ ^44^ ^]^ yet this is something that should be further investigated. Additionally, whether MLO can be found inside leukocyte subsets from other immune tissues is something worth investigating in the future.

In conclusion, we have demonstrated that RMS lesions are characterized by a massive influx of IgM^+^IgD^−^ B cells. We have demonstrated that the IgM^+^IgD^−^ B cells in the RMS lesions are further differentiated toward plasma cells than those of non‐infected fish or even of fish exposed to the infection with no symptomatology. Additionally, we have seen that despite the high levels of IgM in blood found in RMS++ fish, the transcriptional changes observed in spleen and head kidney are faint. Nonetheless, the high percentage of shared clonotypes between skin and head kidney point to some trafficking between these two tissues, which might be responsible for the influx of IgM^+^ B cells in the lesions, given that these cells are not locally proliferating. Finally, the fact that IgM titers in blood are higher precisely in fish with severe symptomatology, and the weak clonal selection in the skin of these fish point to the inefficiency of this exacerbated IgM response in controlling the pathogenesis. The presented data increases our knowledge of RMS, information that will be useful for the future design of novel therapeutic strategies.

## 4 Experimental Section

### Fish

Disease‐free rainbow trout (*Oncorhynchus mykiss*) eyed eggs (sourced from Aquasearch Ova Aps, Billund, Denmark) were hatched and maintained in the high‐contained aquarium facilities at the Technical University of Denmark (DTU). The fish were kept in an aerated recirculating system at 12 ± 1 °C with 12:12 h light/dark cycle. Commercial fish pellets (Biomar A/S, Brande, Denmark) were administered using clock feeders at an appropriate pellet size and rate to match the fish size and temperature. At least a month prior to experimentation, the fish were tagged with a passive integrated transponder (PIT)‐tag in the dorsal musculature under anesthesia to allow individual recognition of the fish. Prior to experimentation, fish were moved to the infection unit and kept under the same conditions as before with the only exception that there was no recirculation of water and all effluent water was let to the sewer after sterilization. The experiments described in the next section were conducted in agreement with Animal Experiments Inspectorate of Denmark (permit no. 2019‐15‐0201‐00159) and approved by the Animal Experiments Committee at DTU.

### Co‐Habitation Challenge and Sampling

An experimental cohabitation model was used to infect naïve fish and induce development of RMS pathology, following the methodology described by Jørgensen et al.^[^ ^33^ ^]^ Here, results from two separate experiments carried out one year apart were presented.

In the first experiment, 4 PIT (passive integrated transponder)‐tagged seeders (394.5 ± 66.8 g and 29.5 ± 4.3 cm) with early RMS pathology were cohabitated with 40 naïve fish (124.8 ± 9.7 g and 21.8 ± 0.6 cm) in a 180 L aquarium in parallel with a control tank containing 50 naïve fish only (124.1 ± 9.7 g and 21.7 ± 0.6 cm). The low seeder to cohabitant ratio ensured low transfer of MLO and low levels of pathology in the cohabitant population. This enabled the comparison of four categories of fish: control fish not exposed to RMS (control), cohabitants with no observable pathology (RMS–), cohabitants with mild pathology (RMS+), and cohabitants with severe pathology (RMS++). The cohabitants were euthanized in an overdose of benzocaine and sampled on two separate days (76 and 78 days post‐cohabitation), which is normally the time when RMS pathology peaks at 12 °C. Over the two days, 7 cohabitants assigned to each of the four categories were sampled.

Due to promising results from the first experiment, the decision was made to expand the scope of analysis in a second experiment. Fish from this second trial were used for all assays in which lesion and non‐lesion sites were compared. For this experiment, 30 seeders (204.2 ± 26.7 g and 25.0 ± 1.2 cm) were cohabited with 120 naïve cohabitants (153.0 ± 30.5 g and 22.6 ± 1.4 cm) in a 600 L aquarium. After 13 days of cohabitation the seeders were removed to a separate aquarium, and the cohabitants were split evenly between two 600 L aquaria. Sixty non‐infected control fish (147.0 ± 29.9 g and 22.3 ± 1.4 cm) were kept under similar conditions in a separate aquarium. For this investigation, a total of 18 fish were sampled: three RMS‐affected fish and three non‐infected control fish on three occasions. The experiment was part of a joint experiment, thus the apparent excess of experimental fish. The RMS‐affected fish sampled on the first two occasions had RMS pathology in early to medium healing stages (the seeders were sampled on these occasions, at which time the seeders were 89 and 101 days post‐cohabitation), while the three RMS‐affected fish on the last occasion had severe active lesions (cohabitants 70 days post‐cohabitation).

Sampling consisted of euthanizing in an overdose benzocaine (Sigma‐Aldrich, Brøndby, Denmark) followed by weighing and measuring. To minimize blood contamination in skin samples, the fish was then bled by complete extraction of blood from the caudal vein plexus using a syringe and needle. Subsequently, samples of skin were collected for isolation of skin leukocytes for flow cytometry, ELISpot, and qPCR analysis. Samples of skin, head kidney, and spleen were dissected and placed into RNAlater (Sigma‐Aldrich) and stored at −80 °C for subsequent qPCR analysis.

### Isolation of Skin Leukocytes

To isolate skin leukocytes, a piece of skin of ≈25 cm^2^ was carefully collected from each side of fish (Figure 1A). To establish if the B cell response was specifically localized in the lesions, in some specific experiments, smaller skin samples were collected from RMS lesions or non‐lesion sites (Figure 1D). In all cases, skin pieces were placed in a Petri dish with 5 ml of Leibovitz's medium (L‐15, Gibco) supplemented with 100 I.U./ml penicillin, 100 µg ml^−1^ streptomycin (P/S, Life Technologies) and 2% fetal calf serum (FCS, Gibco). All muscle tissue was carefully removed, and the skin was cut into small pieces, transferred to a tube containing L‐15 supplemented with P/S, 5% FCS and 2 mg ml^−1^ dispase (Gibco) and incubated for 2 h at 4 °C with continuous agitation. Subsequently, the partly digested skin samples were pushed through a 100 µm nylon cell strainer (BD Biosciences). Samples were then washed by centrifugation (400 x *g* for 15 min) to remove cell debris. Skin cell suspensions were layered onto 30/51% discontinuous Percoll (GE Healthcare) density gradients and centrifuged at 400 x *g* for 30 min at 4 °C, without brake. Cells at the interface, corresponding to leukocytes, were collected and washed in L‐15 containing P/S and 5% FCS. The viable cell concentration was determined by Trypan blue (Sigma‐Aldrich) exclusion and cells were resuspended in L‐15 with 5% FCS at a concentration of 1 × 10^6^ cells/ml.

### Flow Cytometry Analysis

Skin leukocytes were stained with anti‐trout IgM [1.14 mAb mouse IgG1 coupled to R‐phycoerythrin (R‐PE); 0.25 µg ml^−1^],^[^ ^56^ ^]^ anti‐trout IgD [mAb mouse IgG1 coupled to allophycocyanin (APC); 5 µg ml^−1^]^[^ ^57^ ^]^ and anti‐trout MHC II [mAb mouse IgG1 coupled to fluorescein isothiocyanate (FITC); 1.5 µg ml^−1^]^[^ ^58^ ^]^ diluted in staining buffer (phenol red‐free L‐15 medium supplemented with 2% FCS) for 1 h in darkness at 4 °C. All antibodies have been previously characterized and were fluorescently labeled using R‐PE, FITC, or APC Lightning‐Link labeling kits (Innova Biosciences) following the manufacturer's instructions. After staining, cells were washed twice in staining buffer and resuspended in staining buffer for analysis in BD FACS Canto II flow cytometer equipped with BD FACSDiva software (BD Biosciences). The data obtained were analyzed using the FlowJo v.10 software (FlowJo LLC, Tree Star). In all cases, cell viability was checked using 4‐,6‐diamine‐20‐phenylindole dihydrochlorid (DAPI) at 0.2 µg ml^−1^.

### Transcriptional Analysis in Complete Tissues

Total RNA was extracted from skin, spleen and head kidney samples from control non‐infected fish and infected fish (distinguishing between RMS–, RMS+, and RMS++ fish) using TRI Reagent solution (Thermo Fisher Scientific, USA) following the manufacturer´s instructions. For RMS+ and RMS++ fish, skin samples were independently taken from lesion and non‐lesion areas. RNA was quantified by using NanoDrop 1000 Spectrophotometer (Thermo Fisher Scientific) and cDNA was obtained with 1 µg of total RNA using the RevertAid Reverse Transcriptase (Thermo Fisher Scientific) primed with oligo(dT)_23_VN, following manufacturer´s instructions. To evaluate gene transcription levels, LightCycler96 System instrument (Roche) was used for qPCR using FastStart Essential DNA Green Master reagents (Roche) and specific primers (Table S1, Supporting Information). Each sample was subjected to the following conditions: 10 min at 95 °C, followed by 40 amplification cycles (10 s at 95 °C, 10 s at 60 °C and 10 s at 72 °C). A dissociation curve was also obtained by reading fluorescence every degree between 60 °C and 95 °C to ensure only a single product had been amplified. The relative expression levels of the genes were normalized to the expression of *b‐actin*. This reference gene was selected by comparison to other candidates, and after verifying no statistical differences among *b‐actin* Ct values obtained in all samples, following the MIQE guidelines.^[^ ^59^ ^]^ Relative expression levels were calculated by the 2−ΔCt method where ΔCt is determined by subtracting the *b‐actin* value from the target cycle threshold.^[^ ^60^ ^]^ Negative controls with no template and minus‐reverse transcriptase controls were included in all cases.

### Quantification of Serum IgM Titers by ELISA

To determine IgM and IgD levels in serum samples, 96‐well ELISA plates (Greiner) were coated with purified rabbit immunoglobulin to trout IgM^[^ ^61^ ^]^ or with a mixture of six different anti‐light chain (IgL) antibodies (2A1, 2H9, 2D12, 1B4, 3E4 and 1A6), respectively, diluted to 5 µg ml^−1^ in 0.1 m carbonate/bicarbonate buffer pH 9.6. This protocol for detecting total secreted IgD has been previously optimized in rainbow trout by the group.^[^ ^62^ ^]^ The plates were incubated overnight (ON) at 4 °C and then washed 3 times with phosphate buffered saline supplemented with 0.05% Tween 20 (PBS‐T) and blocked with PBS supplemented with 5% skimmed milk and 0.05% Tween 20 (PBS‐T‐SM) (for IgM detection) or PBS supplemented with 1% bovine serum albumin (BSA) and 0.05% Tween 20 (PBS‐T‐BSA) (for IgD detection) for 1 h at room temperature (RT). Thereafter, two‐fold dilutions of trout sera in PBS‐T‐SM (starting from 1/100) for IgM detection or in PBS‐T‐BSA for IgD detection were added to the wells and the plates were incubated ON at 4 °C (for IgM) or 1 h at RT (for IgD). After 3 washes, wells were incubated for 1 h at RT with a mouse mAb to trout IgM (4C10, hybridoma cell culture supernatant diluted 1:50) for IgM detection^[^ ^63^ ^]^ or with biotinylated anti‐trout IgD mAb (1 µg ml^−1^) in PBS‐T‐BSA for IgD detection.^[^ ^62^ ^]^ Following three washes, an HRP‐conjugated rabbit antibody to mouse IgG (Dako P0260) (for IgM) or Streptavidin‐HRP (for IgD) diluted 1:1000 in PBS‐T‐BSA were added to the wells, and the plates were then incubated for 1 h at RT. Finally, after three additional washes, 50 µl of peroxidase substrate (TMB plus, Kem‐En‐Tec Nordic A/S) (for IgM) or 100 µl ml of OPD (O phenylenediamine Dihydrochloride) (Sigma) were added (1 mg ml^−1^) (for IgD) were added to each well. The plates were then incubated at RT for 15 min in the dark. The reaction was stopped by adding 50 µl of sulfuric acid (0.5 N). The plates were read at 450 nm (IgM) or 490 nm (IgD) and at 650 nm (for optical background) in an ELISA reader (Synergy HT, Biotek) and analyzed using the Bio Tek Gen5 software (Agilent). A rainbow trout IgM affinity‐purified from rainbow trout serum using the 4C10 antibody immobilized on agarose beads (Minileak, Kem‐En‐Tec Nordic A/S) was used as a reference. For this, rainbow trout serum diluted 10 times in PBS was pumped through a 5 ml column with immobilized 4C10, and after washing with PBS, bound IgM was eluted with 0.1 m glycin‐HCL pH 2.8. Eluted samples were immediately neutralized in 1 m K_2_HPO_4_ pH 8, and dialyzed back into PBS pH 7.5. The purity of the reference IgM was confirmed by SDS‐PAGE, and the protein concentration was measured using bicinchoninic acid assay (BCA) kit (ThermoFisher) with BSA as reference according to the manufacturer's instructions. All microplate examinations were performed in duplicate wells and the average was used as readout. Positive and negative controls were included in all the plates.

### ELISpot

The number of IgM‐secreting cells in isolated skin leukocytes was determined using ELISpot. For this, ELISpot plates (Millipore) were activated with 70% ethanol and coated with an anti‐IgM mAb (clone 1.14)^[^ ^56^ ^]^ at 2 µg ml^−1^ in PBS and incubated ON in continuous agitation at 4 °C. Before adding the leukocytes, plates were blocked for 2 h at RT with 2% BSA in PBS. The different skin leukocyte suspensions were then added to the wells in duplicate at a concentration of 5 × 10^4^ cells per well. After 24 h of incubation at 20 °C, cells were washed five times with PBS and plates blocked for 1 h at RT with 2% BSA in PBS. After the blocking step, biotinylated anti‐IgM mAb (clone 1.14) was added to the plates at 1 µg ml^−1^ and incubated for 1 h at RT in agitation. Following additional washing steps (five times in PBS), the plates were developed using streptavidin‐HRP (Thermo Scientific) at RT for 1 h in continuous agitation, washed again five times with PBS, and incubated with 3‐amino‐9‐ethylcarbazole (Sigma Aldrich) for 30 min at RT in the dark. The substrate reaction was stopped by washing the plates with water. Once the membranes had dried, they were digitally scanned and the number of spots in each well was determined using an AID iSpot Reader System (Autoimmun Diagnostika GMBH).

### Transcriptional Analysis of Sorted Leukocyte Populations from Skin

Skin IgM^+^ and IgM^−^ cells from control non‐infected fish and infected fish with severe symptomatology (RMS++) were sorted using a FACSAria Fusion sorter equipped with BD FACSDiva software (BD Biosciences). For this, skin leukocytes were stained with anti‐IgM coupled to R‐PE as described above, and IgM^+^ and IgM^−^ cells selected based on the fluorescence emitted and collected (10000 leukocytes from each subset) in staining buffer for subsequent RNA isolation.

In this case, RNA extraction was performed using the Power SYBR Green Cells‐to‐Ct Kit (Invitrogen) following manufacturer´s instructions. DNase treatment and reverse transcription were also performed using the Power SYBR Green Cells‐to‐Ct Kit following the manufacturer's instructions. To evaluate the levels of transcription of the different genes, real time PCR was performed with an Agilent Technologies AriaMX Real‐Time PCR system (AH diagnostics as, Denmark) using 2x Brilliant II SYBR Green qPCR Reagent (Agilent Technologies, Santa Clara, CA, USA) and specific primers (Table S1, Supporting Information). Each sample was measured under the following conditions: 10 min at 95 °C, followed by 40 amplification cycles (15 s at 95 °C and 1 min at 60 °C). The expression of individual genes was normalized to the relative expression of *b‐actin* as described above.

### Immunofluorescence and Confocal Microscopy

Skin tissue obtained from fish with RMS (lesion site) and from fish that have never been exposed to the pathogen (Control) were fixed in 4% paraformaldehyde and processed for paraffin embedding following routine histological procedures. Thereafter, 4 µm thick tissue sections were mounted on Superfrost Plus slides (Menzel‐ Gläser), and double stained using antibodies against rainbow trout IgM and the proliferating cell nuclear antigen (PCNA), an intracellular molecule whose expression and synthesis is linked with cellular proliferation.^[^ ^64^ ^]^ Antigens were retrieved by heating in Tris–EDTA buffer (10 mm Tris base, 1 mm EDTA, pH 9) in a microwave oven for 5 min at 800 W and 5 min at 450 W. Thereafter, non‐specific binding was blocked with 5% BSA in Tris‐buffered saline (TBS). Tissues were then incubated with the primary antibody anti‐trout‐IgM^[^ ^30^ ^]^ diluted 1:10 in blocking buffer (5% BSA/TBS). Incubation with a secondary anti‐mouse IgG1 antibody conjugated with AlexaFluor 488 (ThermoFisher) was followed by further incubation with a mouse IgG2 anti‐PCNA antibody conjugated with AlexaFluor 647 (BioLegend) (diluted 1:100 and 1:50 in blocking buffer, respectively). Sections were then counterstained with DAPI (1 µg ml^−1^, Sigma). All the incubation steps were performed for 1 h at RT in the dark. Tissue autofluorescence was blocked by incubation with 0.3% Sudan black B in 70% ethanol for 10 min, sections were rinsed with TBS and mounted with Fluoromount (Sigma‐Aldrich) for microscopy. Laser scanning confocal microscopy images were acquired with an inverted Zeiss Axiovert LSM 880 microscope with Zeiss Zen software. Tissue images were processed with Zeiss Zen and Adobe Photoshop CS6 software packages.

### IgM Repertoire Analysis

An IgM repertoire analysis was performed in five skin samples from control, RMS– and RMS++ fish. Among the fish used, three fish were selected to also perform an IgM repertoire analysis of their head kidney. In all cases, RNA integrity was determined with a 2100 bioanalyzer using the Eukaryote Total RNA Nano assay (Agilent). Library construction was carried out using a custom protocol as follows. The cDNA synthesis from each sample was performed using 300 ng of total RNA in a reaction including an Oligo‐dT primer (Table S2, Supporting Information) and a template switch oligo (TSO) which included a unique molecular identifier (UMI) (Table S2, Supporting Information) that hybridizes to untemplated C nucleotides added by the reverse transcriptase during reverse transcription. The reaction was performed with SMARTScribe Reverse Transcriptase (Takara Bio) at 42 °C for 90 min followed by incubation with 1 µl of uracil DNA glycosylase (5 U/µl, New England Biolabs) at 37 °C for 40 min. The resultant cDNA was purified using NucleoSpin Gel and PCR Clean‐up (Macherey‐Nagel) at an elution volume of 20 µl. Target enrichment of 5′‐end from IgM encoding transcripts was performed in two rounds of PCR followed by a third PCR for dual‐indexation of PCR products, using in all reactions the Q5 High‐Fidelity DNA Polymerase (New England Biolabs). The first PCR was performed with 1 µl of purified cDNA as template, the primer “Target_Enrichment_FW1” (Table S2, Supporting Information) partially complementary to a region of TSO sequence, and as reverse the primer “IgM_R1” (Table S2, Supporting Information), located in the constant region of IgM, at a final concentration 0.2 µm each. The PCR program was performed for 17 cycles (95 °C for 10 s, 60 °C for 20 s, and 72 °C for 40 s) in a final volume reaction of 25 µl. PCR products were purified using NucleoSpin Gel and PCR Clean‐up (Macherey‐Nagel) at an elution volume of 25 µl. A second PCR was performed with 1 µl of purified PCR product as template, the primer “Target_Enrichment_FW2” (Table S2, Supporting Information) partially complementary to Target_Enrichment_FW1, and as reverse the primer “IgM_R2” located inner in the constant region of IgM which include an additional tail corresponding with first part of P7 illumina adaptor. (Table S2, Supporting Information), both primers at a final concentration 0.2 µm. The PCR program was performed for 13 cycles (95 °C for 10 s, 60 °C for 20 s, and 72 °C for 40 s) in a final volume reaction of 25 µl. PCR product was purified using NucleoSpin Gel and PCR Clean‐up (Macherey‐Nagel) at an elution volume of 25 µl. Finally, 1 µl of purified products from second PCR followed to a PCR round for indexing by which two different 8 nt sample‐specific index, one in both side of amplicons, were incorporated together with the final region of P5 And P7 Illumina adaptors (Table S2, Supporting Information). PCR was performed with sample‐specific primers (final concentration 0.2 µm each) in a final volume reaction of 25 µl. The PCR program was performed for 10 cycles (95 °C for 30 s, 54 °C for 30 s, and 72 °C for 40 s). The PCR results were visualized in a 1.4% agarose gel to corroborate the positive results and the correct size of amplicons. Then, equal volumes (10 µl) of PCR product from each sample was mixed together following with two steps of purification; the first one using NucleoSpin Gel and PCR Clean‐up (Macherey‐Nagel) with NTI buffer diluted 1/3 at an elution volume of 25 µl. This purified product was cleaned and size selected by using SPRIselect beads (Beckman Coulter) at a concentration of 0.6x. At this stage, the ready‐to‐sequencing library was checked using a TapeStation with DNA 1000 kit (Agilent), quantified with the Qubit dsDNA HS Assay Kit (Invitrogen, Life Technologies) and sequenced with NextSeq1000/2000 P1 Reagents (600 cycles) in a NextSeq1000 instrument (Illumina) with protocol 308:8:8:308 cycles and 30% of Phix.

Raw data was demultiplexed and barcodes and sequencing adapters removed using the MIGEC checkout function.^[^ ^65^ ^]^ The first 22 nt from the reverse primer used in the PCRs were used as barcodes for the identification of 3′ ends corresponding to the constant gene of IgM. After a statistical analysis of the molecular identifier group (MIG) by the MIGEC histogram function, an assembly consensus for each sequence was obtained using the MIGEC assemble function. A MIG size threshold of 5, a 1 mm UMI collision filter, and a 1:1 mask was used for processing all samples. Consensus paired reads were merged using the PEAR software,^[^ ^66^ ^]^ with a minimum overlap size of 10 nt. The IgM sequences obtained were compared with the available information from *Oncorhynchus mykiss* contained in the international ImMunoGeneTics information system database^[^ ^67^ ^]^ using the IMGT/HighV‐QUEST tool v1.9.5.^[^ ^68^ ^]^

### Statistical Analysis

All data were analyzed and handled with GraphPad Software (GraphPad Prism v8.0.1, La Jolla California, USA). Prior to analysis, data were checked for normality using Saphiro‐Wilk test. Unpaired or paired two‐tailed Student´s *t*‐test was used in case of normality distributed data, whereas non‐normally data were tested with non‐parametric Wilcoxon matched‐pairs signed‐rank test or non‐parametric Mann‐Whitney test for unpaired data. To compare the transcriptional profile of skin, spleen and head kidney, an ANOVA was performed and significantly different values in each group were indicated by different lower‐case letter. Also, VDJ configuration in the skin was analysed by an ANOVA, followed by a Tukey's HSD post‐hoc test when data were normally distributed, or evaluated by a non‐parametric Kruskal‐Wallis test when they were not normal. Data were presented as mean + SEM or mean + SD, and significance between means was stablished at *p* ≤ 0.05, indicating in some cases different degrees of significance (* *p* ≤ 0.05, ** *p* ≤ 0.01 and *** *p* ≤ 0.001).

### Ethics approval statement

The experiments described were conducted in agreement with Animal Experiments Inspectorate of Denmark (permit no. 2019‐15‐0201‐00159) and approved by the Animal Experiments Committee at DTU.

## Conflict of Interest

The authors declare no conflict of interest.

## Author Contributions

C.T., J.G.S., and N.L. conceived the study. J.G.S. performed most of the fish infection experimental work with help from S.V.‐G., while J.G.H.‐J. and S.V.‐G. performed most of the lab experimental work, with help from J.G.S. E.M. performed most of the flow cytometry analysis from this study, with help from D.M. P.J.‐B. and P.P. performed and analyzed the IgM repertoires with technical help from M.V.‐R. R.S. performed the confocal analysis. C.T. and J.G.H.‐J. wrote the main body of the manuscript with help and contributions from all other authors. All authors read and approved the final version of the manuscript.

## Supporting information



Supporting Information

## Data Availability

The data that support the findings of this study are available from the corresponding author upon reasonable request.
