# Supplementary material for: Characterization of B Cell Responses in Rainbow Trout (Oncorhynchus Mykiss) Affected by Red Mark Syndrome
Source: Adv Sci (Weinh). 2025 Jun 25;12(35):e03047. doi: 10.1002/advs.202503047 (PMC12462978; doi:10.1002/advs.202503047)
Supplement: Supplementary file 1 — Supporting Information [file ADVS-12-e03047-s001.pdf]

ADVANCED SCIENCE
Open Access

Supporting Information

for *Adv. Sci.*, DOI 10.1002/advs.202503047

Characterization of B Cell Responses in Rainbow Trout (*Oncorhynchus Mykiss*) Affected by Red Mark Syndrome

J Germán Herranz-Jusdado, Samuel Vicente-Gil, Esther Morel, Pablo Jiménez-Barrios, Rocío Simón, Pedro Perdiguero, Diana Martín, Marta Vargas-Ramírez, Niels Lorenzen, Jacob Günther Schmidt and Carolina Tafalla**

Table S1. List of primers used for real time PCR analysis of gene expression in this study

Gene name	Forward primer (5´-3´)	Reverse primer (5´-3´)
b-actin	TCCTTCCTCGGTATGGAGTCT	TTACGGATGTCCACGTCACAC
membrane *igm*	CCTACAAGAGGGAGACCGATTGTC	GTCTTCATTTCACCTTGATGGCAGT
secreted *igm*	TACAAGAGGGAGACCGGAGGAGT	CTTCCTGATTGAATCTGGCTAGTGGT
membrane *igd*	TGAACATATCCAAACCAGAGCTCC	GTCCTGAAGTCATCATTTTGTCTTGA
secreted *igd*	TGAACATATCCAAACCAGGTGTCTG	GTCCTGAAGTCATCATTTTGTCTTGA
membrane *igt*	TCGAAGTCCACGGCGAACA	GTGTTCTTCACCGCTTCATCTTGAA
secreted *igt*	CATCAGCTTCACCAAAGGAAGTGA	TCACTTGTCTTCACATGAGTTACCCGT
prdma1a-1	CAGCGCCCCAGTCAAGATA	GGGGGTAGAGGGCACAGC
prdma1a-2	CATTCGGCCCTATGTGTGG	CCCCTCGGTAGTCAACATGG
prdma1c-1	TCACTGCATCAACACCGAGA	CCGGTCTCCATCACCATCTT
prdma1c-2	CGCCAATGGGAATATGTCA	GACATAGCCAGGATGCAGA
irf4	CGCATCACCATAGCAACACC	CTCCTCTCCCCAGGCTTTCT
bcma	ATGTCAGAAGGACAGTGTGGACTGG	CGGCTCTGGGGCTTTGCTCT
pax5	ACGGAGATCGGATGTTCCTCTG	GATGCCGCGCTGTAGTAGTAC
mlo	GCGGTTATCTGGGCAGTC	TGCGACACGAAACCTAAG
pcna	CCGACATGGGCCACGTTAAG	ACAGGAAGTAGGAACCAACCCC

Table S2. Primers used in different steps of the library constructions for reperoire analysis

Primer	Sequence (5´- 3´)
	**cDNA synthesis**
**oligodT**	AAGCAGTGGTATCAACGCAGAGTACTTTTTTTTTTTTTTTTTTTTTTTTTTTTTTVN
**TSO_UMI**	CTACACGACGCUCTTCCGAUCTUNNNNUNNNNUNNNNUCTTrGrGrGrG*
	**Target Enrichment PCR 1**
**Target_Enrichment_FW1**	CTACACGACGCTCTTCCGATCT
**IgM_R1**	AAAGTCATTGGCAAAGCAGG
	**Target Enrichment PCR 2**
**Target_Enrichment_FW2**	CTCTTTCCCTACACGACGCTC
**IgM_R2**	GTGACTGGAGTTCAGACGTGTGCTCTTCCGATCTCCAGAGTCATCATATCTCCGGT
	**Index PCR**
**P5_index**	AATGATACGGCGACCACCGAGATCTACACXXXXXXXXACACTCTTTCCCTACACGACGCTC
**P7_index**	CAAGCAGAAGACGGCATACGAGATCGTGATXXXXXXXXGTGACTGGAGTTCAGACGTGT

V and N – IUPAC codes; **rG – RNA base**; **U** – DNA base
